# Supplementary figures and images for: Antigen receptor control of methionine metabolism in T cells
Source: eLife. 2019 Mar 27;8:e44210. doi: 10.7554/eLife.44210 (PMC6497464; doi:10.7554/eLife.44210)

Figure 1

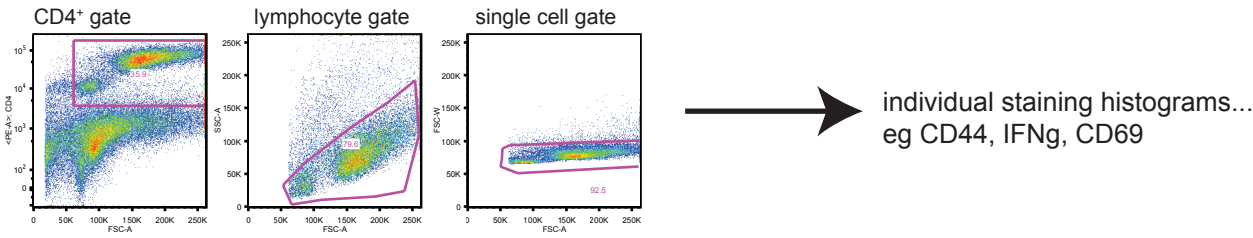

Figures 1 and 2

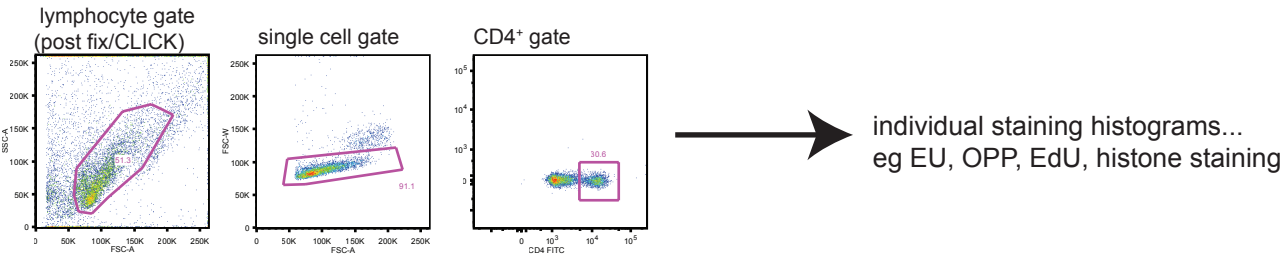

Figure 2

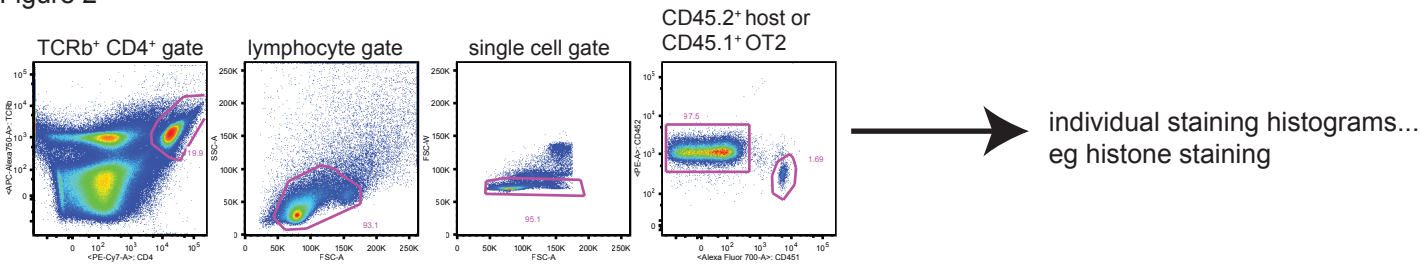

Supplement: Supplementary file 1. [file elife-44210-supp1.pdf]
